# Supplementary material for: Additional energy scale in SmB6 at low-temperature
Source: Nat Commun. 2016 Dec 12;7:13762. doi: 10.1038/ncomms13762 (PMC5159841; doi:10.1038/ncomms13762)
Supplement: Supplementary Information — Supplementary Figures 1-7 and Supplementary Notes [file ncomms13762-s1.pdf]

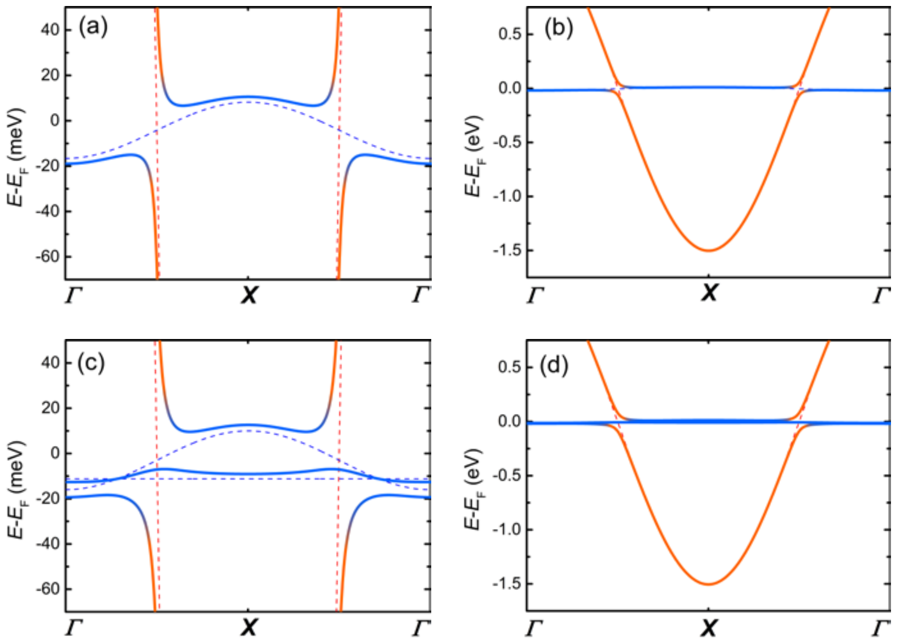

Supplementary Figure 1 | Simplified schematic of the hybridization in the Kondo lattice of SmB_6. (**a**) and (**b**) We assume one *f*-band hybridizing with the *d*-band, an effect which is normally used to interpret the experimental data. (**c**) and (**d**) A more realistic model with two *f*-bands is employed. The gap-opening process is shown from *Γ* to *X* point. **a** and **c** are zoomed plots of **b** and **d** around the Fermi energy. Blue and red colors indicate the ratio of *f*- and *d*-electron character in the bands, respectively. ARPES measurements confirmed a highly dispersive *d*-band (ε_d) centered around the *X* point, and three flat *f*-bands located around -15 meV, as well as -160 meV, and -960 meV (not shown). As depicted in Fig. 1 of the main text, the *f*-state at around -15 meV is further split into three nearly degenerate sub-*f*-bands: Γ_7, $\Gamma_8^{(1)}$, and $\Gamma_8^{(2)}$. In principle, only Γ_7 and $\Gamma_8^{(1)}$ bands hybridize with the *d*-band since they possess the same symmetry. For simplicity, we leave out the $\Gamma_8^{(2)}$ band and other *f*-bands, the latter of which are far away from E_F. To simulate the gap-opening process, simple mean-field Hamiltonians are used:

$$\mathcal{H}_1 = \begin{bmatrix} \varepsilon_d & \nu \\ \nu & \Gamma_7 \end{bmatrix} \quad \text{and} \quad \mathcal{H}_2 = \begin{bmatrix} \varepsilon_d & \nu & \nu \\ \nu & \Gamma_7 & 0 \\ \nu & 0 & \Gamma_8^{(1)} \end{bmatrix}.$$

Here, $\mathcal{H}_1$ corresponds to the case where one *f*-band hybridizes with one conduction band while $\mathcal{H}_2$ describes the case containing two *f*-bands and one conduction band. The values for ε_d can be determined *e.g.* by ARPES measurements. For the *f*-bands we used a similar expression and parameters as given in Ref. 1. Based on this reference, we model the bands by:

$$\varepsilon_d = 2t_d(\cos(k_x-\pi)+\cos(k_y)+\cos(k_z)) - \mu_d,$$

$$\Gamma_7 = 2t_{f1}(\cos(k_x-\pi)+\cos(k_y)+\cos(k_z)) + 4r_{f1}(\cos(k_x-\pi)\cos(k_y)\cos(k_z)) - \mu_{f1},$$

$$\Gamma_8^{(1)} = 2t_{f2}(\cos(k_x-\pi)+\cos(k_y)+\cos(k_z)) + 4r_{f2}(\cos(k_x-\pi)\cos(k_y)\cos(k_z)) - \mu_{f2}.$$

Here, the parameters *t* and *r* describe the dispersion of the respective bands as indicated by the subscripts d, f1 and f2, and μ are the respective chemical potentials. We set t_d = -750 meV, μ_d = 3000 meV, t_{f1} = 0.5 meV, r_{f1} = 3 meV, μ_{f1} = -5 meV, t_{f2} = 0.2 meV, r_{f2} = 0.1 meV, μ_{f1} = -12 meV. It is noted that the hybridization amplitude is assumed to be the same for all *d*-*f* hybridizations, *i.e.* ν = 60 meV, and the interaction between *f*-bands is set to zero for simplicity. By diagonalizing these Hamiltonians, the main features of the hybridization gap-opening process can essentially be captured. In this simulation, we assume $\Gamma_8^{(1)}$ to be closer to E_F than Γ_7 at the *Γ* point, but the reversed assignment of these two multiplets has little impact on the hybridized band structure. In particular, the finding of an additional energy scale is not influenced by this assumption.

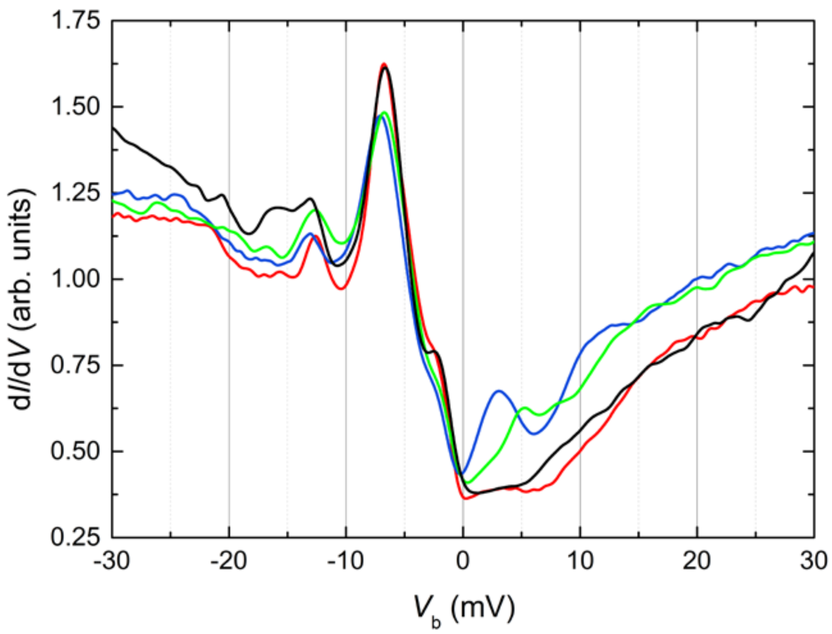

Supplementary Figure 2 | Variation of tunneling conductance spectra. *g*(*V*)-curves measured at different clean and non-reconstructed (001) surfaces of SmB_6 at 0.35 K (V_b = 50 mV, I_{sp} = 150 pA, V_{mod} = 0.3 mV). Those spectra which were measured on the same cleaved surface were obtained at positions spaced more than 100 nm apart. In particular, the features at about ±3 mV show clear spatial dependence.

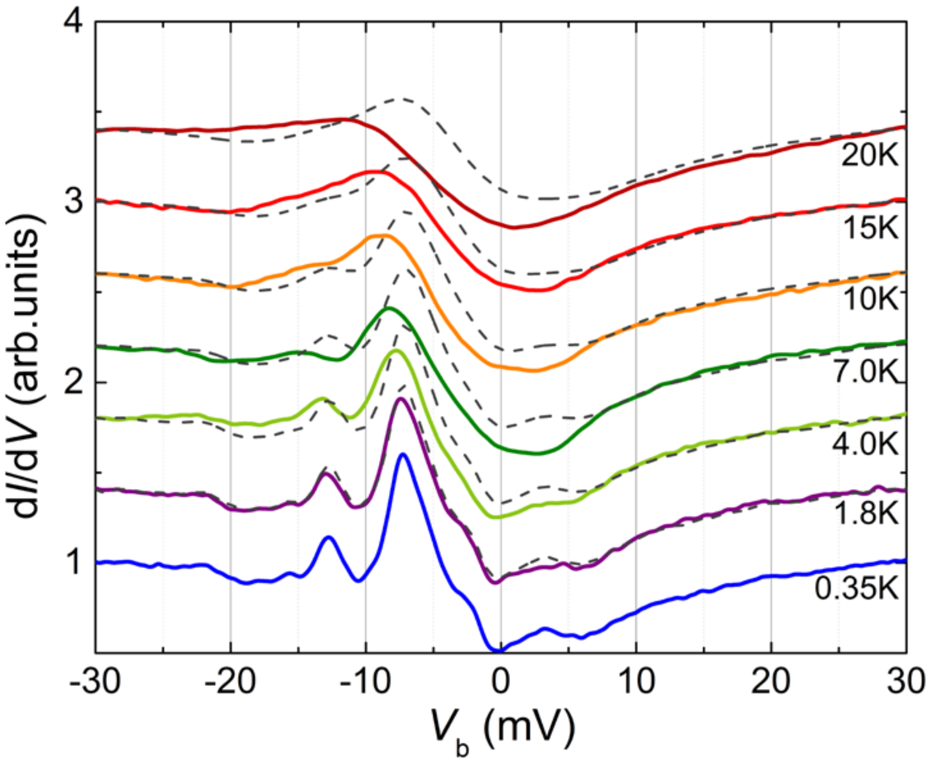

Supplementary Figure 3 | Influence of thermal broadening. The spectra measured at different temperatures (solid lines) are compared to the calculated data considering thermal broadening (gray dashed lines) based on the d*I*/d*V*-curve obtained at 0.35 K (V_b = 30 mV, I_{sp} = 100 pA, V_{mod} = 0.3 mV). The experimental data exhibit a much stronger temperature dependence than expected from thermal broadening only. Curves are equally shifted for clarity.

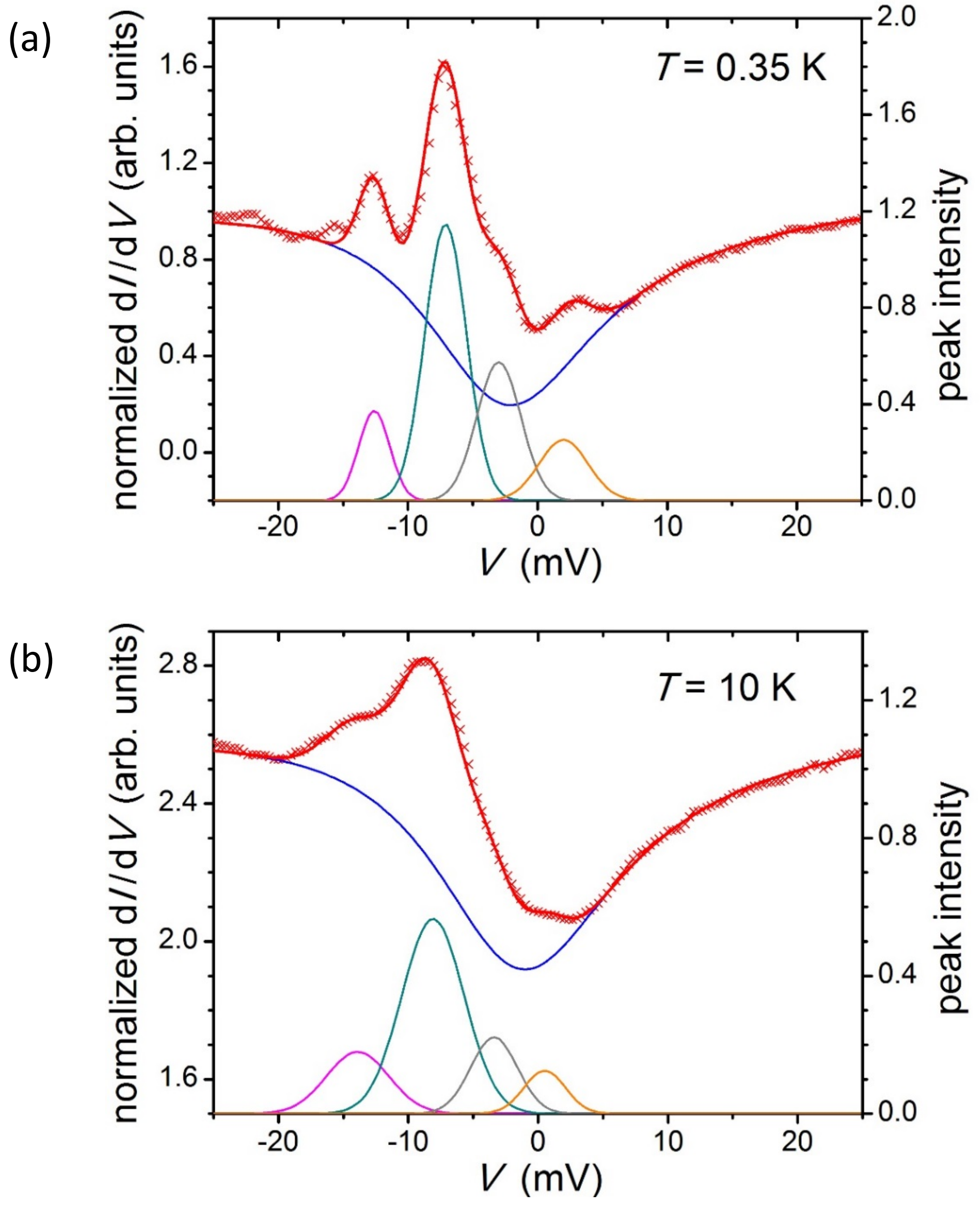

Supplementary Figure 4 | Attempts to fit the experimental spectra. (**a**) Representative fit (red lines) to the d*I*/d*V*-data (red crosses) obtained at 0.35 K using a Fano model (blue line) and four Gaussian peaks (right scale). (**b**) The same fit procedure is applied to the tunneling conductance data at 10 K. The parameters used are:

T	E_0 (meV)	Γ (meV)	$\lvert q \rvert$	peak positions (mV)	Gaussian widths (mV)
0.35 K	-3	16.5	0.12	-12.6; -7.1; -3.2; 2.0	1.2; 1.5; 1.8; 1.9
10 K	-2	17.5	0.12	-13.9; -8.1; -3.4; 0.5	1.1; 3.4; 1.0; 0.5

(see Supplementary Figure 5 for the Fano model). Albeit the fits nicely reflect the data, a range of parameters can be used. Specifically at higher temperature T = 10 K, varying the parameters E_0 and Γ of the Fano model changes the Gaussian peak height drastically while keeping the quality of the fit. This renders the fits at such higher temperature unreliable. We note that a single Gaussian peak can be used at even higher temperatures, T = 20 K, providing more reliable results (see Supplementary Figure 5). This may explain the good agreement of the data obtained by Ruan *et al.*, Ref. 1.

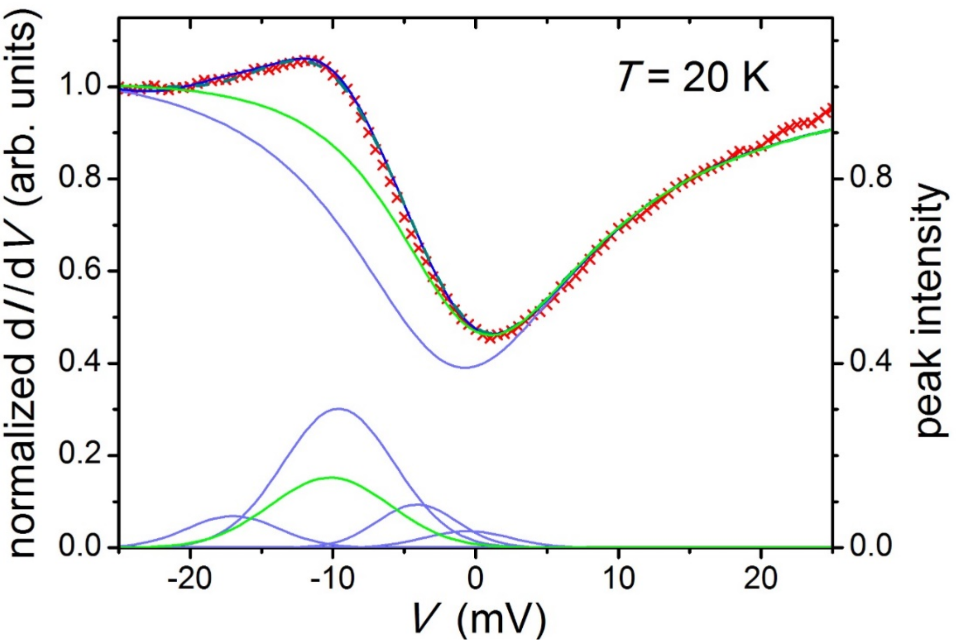

Supplementary Figure 5 | Ambiguity of the fit to data obtained at high temperature. Tunneling conductance measured at T = 20 K (red crosses). These data can be fitted by the Fano formula: $g(\mathrm{V}) \propto (\varepsilon + q)^2/(\varepsilon^2 + 1)$, where $\varepsilon = 2(\mathrm{e}V - E_0)/\Gamma$. Here, Γ describes the resonance width and q is related to the tunneling ratio between the 4f states and the conduction band as well as the particle-hole asymmetry of the conduction band. Such a fit yields Γ = 15.7 meV and q = 0.58, in agreement with Ref. 2. A value q < 1 is to be expected for a B-terminated surface as investigated here. However, also a fit including a contribution from a single Gaussian peak (located at -10.2 mV and with half-width σ = 4.2 mV, bottom green line and right scale) represents the tunneling conductance data well (dark cyan line), being similar to the procedure applied in a previous report [1]. Our low-temperature measurements (Supplementary Figure 4) suggest that a single Gaussian does not suffice to describe the data and hence, four Gaussian peaks were used to describe the data at 0.35 K, *cf*. Supplementary Figure 4a above. As one example, using four Gaussians (bottom light-blue lines) on top of one particular Fano lineshape (upper light blue line) reflects the measured $g(V)$ data also very well (dark blue line). We note that the different fits can barely be distinguished and a broad range of parameters can be employed at comparatively high temperatures T = 20 K (in particular for the description of the underlying hybridization). For this reason we refrained from detailed fits and rather used the peak intensity of the normalized spectra for our analysis, see Fig. 4e of the main text.

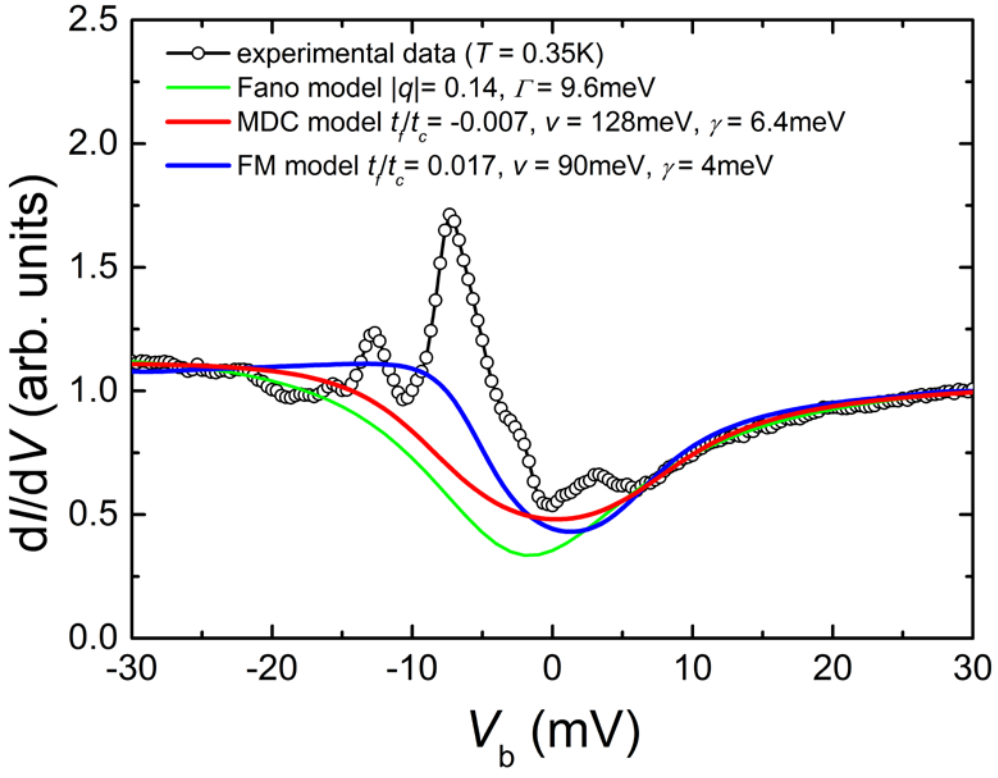

Supplementary Figure 6 | Simulating the bulk Kondo hybridization gap of SmB_6 at 0.35 K using different models. Comparison of experimental data to the Fano model (Ref. 3), Maltseva, Dzero and Coleman's model (MDC model, Ref. 4) as well as Figgins and Morr's model (FM model, Ref. 5). As mentioned in the main text, our spectra measured at different T overlap nicely for $V_b < -25$ mV and $V_b > 10$ mV, so this voltage range was used primarily for the fittings. Details of these models can be found in previous reports, Refs. 1, 6. For comparison, we kept the band structure-related parameters the same as the ones used by Yee *et al.*, Ref. 6, but left the tunneling ratio t_f/t_c, the quasiparticle scattering rate γ and the hybridization strength v as free parameters in the MDC and the FM models. We simplified the models by taking the quasiparticle scattering rates identical for the f-electrons and the conducting electrons. The position of the f-band E_0^f was chosen to -3 meV for all models. The best fitting parameters are listed in the figure and compare to those obtained in previous reports.

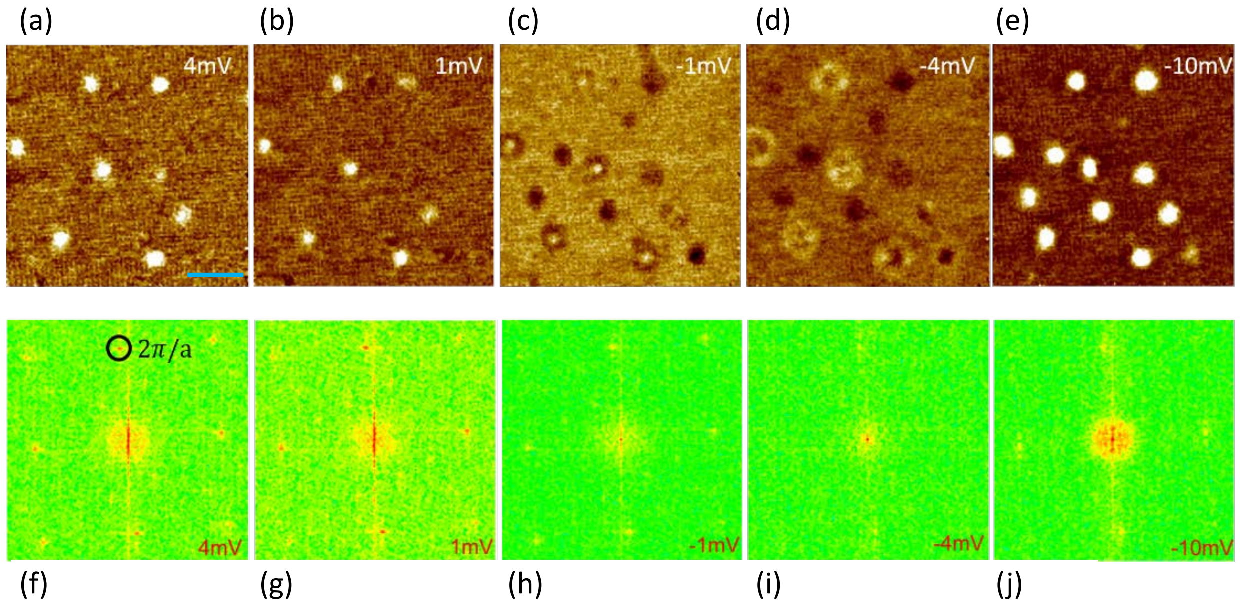

Supplementary Figure 7 | Attempt towards quasiparticle interference (QPI) on SmB_6. Here, the same B-terminated surface is investigated as shown in Fig. 2b of the main text (scale bar 5 nm). (**a**) – (**e**) Some selected d*I*/d*V*-maps taken at the indicated bias voltages V_b over an area of 20 nm x 20 nm with a resolution of 128 x 128 pixels and at a temperature T = 1.8 K. Even though the majority of the defects can be recognized at all V_b, some of them are not visible at positive V_b. This points towards a different nature of these defects. (**f**) – (**j**) Corresponding Fourier transforms of the d*I*/d*V*-maps. The good quality of our surface is reflected in the presence of lattice peaks at 2π/a. We focus here on low energies in accord with measurements by ARPES, Ref. 7, and on planar tunnel junctions, Ref. 8. No clear evolution of any QPI pattern is observed, similar to earlier results in Ref. 1. Based on the negligible magnetic field response, we speculate that most impurities are likely nonmagnetic. In this case, spin-dependent or spin-conserving scattering dominates, Ref. 9. If we assume that SmB_6 is indeed a topological Kondo insulator, the scattering amplitude is expected to be suppressed as states at $\boldsymbol{k}$ and $-\boldsymbol{k}$ have opposite spin directions on the Dirac cone. In this case, the absence of clear QPI signatures is consistent with topologically protected surface states while it may render a scenario based on mere Rashba splitting less likely, see Ref. 9.

Supplementary References

1. Ruan, W. *et al*. Emergence of a Coherent In-Gap State in the SmB_6 Kondo Insulator Revealed by Scanning Tunneling Spectroscopy. *Phys. Rev. Lett.* **112**, 136401 (2014).
2. Rößler, S. *et al*. Hybridization gap and Fano resonance in SmB_6. *Proc. Natl. Acad. Science USA* **111**, 4798-4802 (2013).
3. Fano U. Effects of Configuration Interaction on Intensities and Phase Shifts. *Phys. Rev*. **124**, 1866-1878 (1961).
4. Maltseva, M., Dzero, M. & Coleman, P. Electron tunneling into a Kondo lattice. *Phys. Rev. Lett*. **103**, 206402 (2009).
5. Figgins, J. & Morr, D. K. Differential Conductance and Quantum Interference in Kondo Systems. *Phys. Rev. Lett*. **104**, 187202 (2010).
6. Yee, M. M. *et al*. Imaging the Kondo Insulating Gap on SmB_6. Preprint at http://arxiv.org/abs/1308.1085 (2013).
7. Neupane, M. *et al*. Surface electronic structure of the topological Kondo-insulator candidate correlated electron system SmB_6. *Nat. Commun*. **4**, 2991 (2013).
8. Park, W. K., Sun, L., Noddings, A., Kim, D.-J., Fisk, Z. & Greene, L. H. Topological surface states interacting with bulk excitations in the Kondo insulator SmB_6 revealed by planar tunneling spectroscopy. *Proc. Nat. Acad. Sci. USA* **113**, 6599-6604 (2016).
9. Oka, H., Brovko, O. O., Corbetta, M., Stepanyuk, V. S., Sander, D. & Kirschner, J. Spin-polarized quantum confinement in nanostructures: Scanning Tunneling Microscopy. *Rev. Mod. Phys*. **86**, 1127-1168 (2014).
